# „Hallesche OP-Wochen“: Wie ein Lehrformat das Interesse von Medizinstudierenden an der Chirurgie weckt

**DOI:** 10.1007/s00104-020-01281-w

**Published:** 2020-09-18

**Authors:** Elisa Haucke, Katharina Clever, Stefan Watzke, Johanna Schubert, Dietrich Stoevesandt, Christiane Ludwig, Sebastian Plößl, Stefan K. Plontke

**Affiliations:** 1 Dorothea-Erxleben-Lernzentrum, Medizinische Fakultät, Martin-Luther-Universität Halle-Wittenberg, Halle (Saale), Deutschland; 2 Universitätsklinik und Poliklinik für Hals-Nasen-Ohren-Heilkunde, Kopf- und Hals-Chirurgie, Universitätsklinikum Halle, Ernst-Grube-Str. 40, 06120 Halle (Saale), Deutschland; 3 Klinik und Poliklinik für Psychiatrie, Psychotherapie und Psychosomatik, Universitätsklinikum Halle, Halle (Saale), Deutschland

**Keywords:** Lehrmethoden, Liveoperation, Live-Teaching, Medizinstudium, Chirurgische Ausbildung, Teaching methods, Live surgery, Live teaching, Medical studies, Surgical training

## Abstract

**Hintergrund:**

Eine studierendenorientierte Ausbildung ist in der praktischen Chirurgie schwierig und nur unzureichend umsetzbar. Neue Lehrkonzepte sind notwendig, um Studierenden die Chirurgie näherzubringen und sie für das Fach zu begeistern.

**Methodik:**

In einer zweiwöchigen fakultativen Veranstaltung konnten Studierende der Humanmedizin insgesamt acht chirurgische Eingriffe aus verschiedenen Fachrichtungen live im Hörsaal mitverfolgen. Zeitgleich erfolgte im Hörsaal eine Moderation durch eine/n erfahrene/n Chirurgen/in. Anhand von Fragebögen (prä/post) wurde begleitend zu jeder Operation untersucht, inwieweit sich die Lehrveranstaltung eignet, das Verständnis und das Interesse für die chirurgische Tätigkeit zu verbessern.

**Ergebnisse:**

Insgesamt lagen 709 vollständige Tagesevaluationen (prä und post) von 381 Studierenden vor. Der selbstberichtete Lerneffekt wurde von den Studierenden als gut bis sehr gut bewertet. In vielen dargestellten Fachgebieten zeigten sich durch die Teilnahme an den Liveoperationen signifikant positive Einstellungsänderungen in Bezug auf das jeweils operierende Fach allgemein, in Bezug auf eine geplante Famulatur und für die Wahl als späteres Fachgebiet.

**Schlussfolgerung:**

Die hohen Teilnehmendenzahlen und die Evaluationsergebnisse lassen auf eine hohe Akzeptanz der Lehrveranstaltung schließen. Das Lehrformat ist geeignet, das Verständnis für chirurgische Abläufe zu verbessern sowie die Einstellung von Medizinstudierenden gegenüber chirurgischen Fachgebieten positiv zu beeinflussen.

## Abstract (en)

**Background:**

A practical education in surgery is difficult to conduct in a student-centered manner and is thus often inadequate. New teaching concepts are required to provide students with insights into surgery and make the field more appealing.

**Methods:**

As part of a two-week facultative event, medical students followed eight live surgical procedures from different disciplines from an auditorium. In the auditorium, the procedures were simultaneously moderated by an experienced surgeon. Before and after every procedure, questionnaires were used to analyze whether the teaching event was suited to improve an understanding and interest for the field of surgery.

**Results:**

A total of 709 completed questionnaires (pre and post) from 381 students were collected. The self-reported learning effect was evaluated as good or very good by students. In many of the presented disciplines, experiencing a live surgical procedure was associated with significant positive changes in attitude regarding each discipline in general, a potential clinical traineeship in the discipline and choosing the discipline as a later specialization.

**Conclusion:**

The high attendance as well as the evaluation results suggest a high acceptance for the teaching event. The teaching format is suited to improve the understanding of surgical procedures and had a positive effect on medical students’ attitude towards surgical disciplines.

Medizinstudierende messen den chirurgischen Fertigkeiten und dem Verständnis für die chirurgischen Prinzipien eine große Bedeutung für ihre spätere Tätigkeit zu. Die Ausbildung in diesen Fertigkeiten wird jedoch als inadäquat eingeschätzt [1]. Erhebungen unter Medizinstudierenden prognostizieren, dass am Ende des Studiums nur ca. 10 % der Studierenden eine chirurgische Weiterbildung anstreben [7]. Die vorliegende Studie zeigt, dass das Lehrformat „Hallesche OP-Wochen“ das Verständnis für chirurgische Abläufe verbessert und die Einstellung gegenüber chirurgischen Fachgebieten positiv beeinflusst.

## Hintergrund und Fragestellung

Eine studierendenorientierte Ausbildung ist in bestimmten klinischen Bereichen schwierig oder nur unzureichend umsetzbar, da Zeit- und personeller Mangel den Klinikalltag prägen [9, 10]. Aufgrund begrenzter räumlicher Kapazitäten und Hygienevorschriften ergeben sich zudem Einschränkungen für die praktische Ausbildung im Operationssaal. Neue Lehrkonzepte sind notwendig, um mit den gegebenen Umständen eine qualitativ hochwertige, praktisch orientierte und studierendenzentrierte Ausbildung zu gewährleisten und für die Chirurgie zu begeistern.

Die Liveübertragung oder Videoaufzeichnung chirurgischer Eingriffe ist durch den Fortschritt der Technik eine immer beliebtere Methode, um die operative Medizin abzubilden. Oft kommt dieses Lehrformat allerdings erst in der Weiterbildung zum/r Facharzt/ärztin zum Einsatz und umfasst wenige Lehreinheiten aus einzelnen Fachbereichen [4, 5, 8]. Endoskopien, Laparoskopien und roboterassistierte Operationen werden dabei häufig angeboten, da sich die Eingriffe technisch komplikationslos übertragen lassen [2].

Die „Halleschen OP-Wochen“ stellen ein Novum in der Liveübertragung realer Operationen und chirurgischer Eingriffe für Studierende dar. In einer zweiwöchigen Veranstaltung präsentieren verschiedene chirurgische Fachrichtungen des Universitätsklinikums Halle (UKH) „klassische“, d. h. für das chirurgische Fachgebiet typische und häufig durchgeführte Operationen oder Interventionen im Bereich der Inneren Medizin oder interventionellen Radiologie. Die geplanten Eingriffe werden im Vorfeld online bekannt gegeben, um den Studierenden die Möglichkeit zu geben, sich mit den jeweiligen Operationstechniken und Krankheitsbildern vertraut zu machen. Neben der moderierten Liveübertragung der Operation in Hörsäle wird durch ergänzende Hintergrundinformationen vertiefend auf das jeweilige Krankheitsbild einschließlich Anatomie, (Patho‑)Physiologie und das Operationsverfahren eingegangen. Damit wird neben der curricularen Lehre (Blockpraktikum, Seminar und Vorlesung) unterstützend ein Teil der im Nationalen Kompetenzbasierten Lernzielkatalog Medizin (NKLM) festgelegten Lernziele abgebildet (Kap. 16: Therapeutische Prinzipien, Kap. 21: Erkrankungsbezogene Prävention, Diagnostik, Therapie, Versorgungs- und Notfallmanagement) und die Möglichkeit geboten, häufige Therapieverfahren kennenzulernen. In den curricularen Lehrveranstaltungen ist dafür nur begrenzt Zeit zur Verfügung und vor allem den Famulaturen/dem praktischen Jahr vorbehalten.

Die vorliegende Studie untersuchte, inwieweit sich die „Halleschen OP-Wochen“ eignen, das Interesse von Medizinstudierenden an chirurgischen Fächern zu steigern. Dafür wurden der selbstberichtete Lerneffekt und die Einstellung der Studierenden gegenüber den einzelnen chirurgischen Fachgebieten untersucht. Um die Akzeptanz des Lehrformates zu evaluieren, wurden darüber hinaus die Bewertung von Struktur und Organisation der Veranstaltung analysiert und Teilnehmendenzahlen ausgewertet.

## Studiendesign und Untersuchungsmethoden

### Ablauf der „Halleschen OP-Wochen“

Im Regelbetrieb der einzelnen Kliniken des UKHs gibt es an unterschiedlichen Tagen der Woche sog. „lange Tische“ für komplexe und zeitintensive Operationen mit einem zugeteilten Operationsteam. Im Rahmen der „Halleschen OP-Wochen“ wird dies genutzt, damit an acht Abenden (jeweils 18:00 bis 20:00 Uhr über zwei Wochen) acht ausgewählte chirurgische Fachrichtungen den Studierenden eine für ihr Fachgebiet typische Operation vorstellen können (Tab. 1).

**Table 1:** 

Tag	Fachgebiet	Operation	(*n*)
1	Allgemeinchirurgie	Cholezystektomie	48
2	Orthopädie	Knieendoprothese	63
3	Neurochirurgie	Vestibularisschwannom	110
4	Hals-Nasen-Ohren-Heilkunde	Cochleaimplantat	93
5	Traumatologie	Ersatzplastik vorderes Kreuzband	83
6	Gynäkologie	Laparoskopische Hysterektomie	76
7	Herz-Thorax-Chirurgie	Bypassoperation	150
8	Urologie	Tumornephrektomie	86
			*709*

*n* Anzahl der Teilnehmenden mit vollständiger Tagesevaluation (Prä- und Post-Fragenbogen)

**Figure 1:**
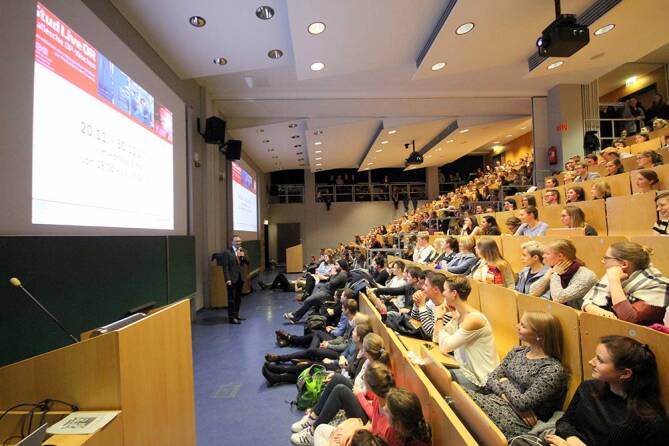

Die Teilnahme war für die Studierenden fakultativ. Die Information über die „Halleschen OP-Wochen“ wurde über die Lehrveranstaltungen, die Fachschaft Medizin und die sozialen Netzwerke der Studierenden der Universitätsmedizin Halle verbreitet. Vor jeder Operation wurde das schriftliche Einverständnis des/der Patienten/in für die Liveübertragung eingeholt. Im Operationssaal wurden zusätzlich zu den je nach Fachrichtung und Operation verwendeten Kamerasystemen (Operationsmikroskope und Endoskope) allgemeine Saalkameras installiert. Der/die Operateur/in wurde mit einem Headset ausgestattet. Die visuelle und verbale Liveübertragung der Operationen erfolgte in bis zu vier miteinander verbundene Hörsäle des UKH (Abb. 1).

Im Hörsaal wurden die Operationen von einem/r erfahrenen Chirurgen/in des jeweiligen Fachgebietes als Moderator/in begleitet, fachlich eingebettet und erläutert. Dies erfolgte in freiwilliger Unterstützung außerhalb der Dienstzeit. Der Ablauf der Veranstaltung war wie folgt gegliedert:

1. Fallvorstellung,
2. generelle Aspekte zum Krankheitsbild und zu den Therapieprinzipien (z. B. Kernpunkte für Prüfungen/Staatsexamen/die ärztliche Praxis),
3. Beschreibung der Anatomie und der Operationstechnik,
4. Liveübertragung aus dem Operationssaal.

Während der Operation erfolgte eine Erklärung der einzelnen Arbeitsschritte durch den/die Operateur/in. Die Studierenden konnten live Fragen stellen, die entweder von den Moderatoren/innen im Hörsaal oder dem/der Operateur/in direkt aus dem Operationssaal beantwortet wurden.

### Datenerhebung

Im ersten Jahr der „Halleschen OP-Wochen“ (2014) erfolgte vor und nach jeder einzelnen Operation eine quantitative, anonymisierte Datenerhebung mittels selbstkonstruierter Fragebögen. Für die Zuordnung der Prä- und Post-Fragebögen einer Person generierten die Teilnehmenden einen 8‑stelligen Teilnahmecode.

Neben soziodemografischen Angaben (Alter, Geschlecht, aktuelles Studienjahr) wurde die Beurteilung der (1) **Struktur und Organisation der Veranstaltung** auf einer 6‑stufigen Skala im Schulnotensystem von 1 = „sehr gut“ bis 6 = „ungenügend“ erfragt (Post-Fragebogen). Auf einer 5‑stufigen Skala (1 = „trifft völlig“ zu, 5 = „trifft gar nicht zu“) wurde zudem der (2) **selbstberichtete Lerneffekt** der Studierenden durch die folgenden Aussagen erfasst (Post-Fragebogen): *Die Veranstaltung war geeignet, mein Verständnis für den Ablauf der Operation zu verbessern und *Die Veranstaltung war geeignet, mein Verständnis der zugrunde liegenden Erkrankung zu verbessern.

Zur Erfassung der (3) **Einstellung zu den jeweiligen chirurgischen Fachgebieten **bewerteten die Studierenden (Prä- und Post-Fragebogen) die folgenden Aussagen auf einer 5‑stufigen Skala (1 = „trifft völlig zu“, 5 = „trifft gar nicht zu“) am Beispiel der Allgemeinchirurgie: *Allgemeinchirurgie finde ich spannend, *Ich kann mir eine Famulatur in der Allgemeinchirurgie gut vorstellen und *Ich kann mir Allgemeinchirurgie als späteres Fachgebiet gut vorstellen. Die Aussagen wurden jeden Tag für das jeweilige chirurgische Fachgebiet neu formuliert.

### Datenanalyse

Die Auswertung der Fragebögen erfolgte mithilfe des Statistikprogramms IBM SPSS Statistics (Version 25). Ein α‑Niveau von 0,05 wurde für alle Berechnungen festgelegt. Charakteristika der Stichprobe, Struktur und Organisation der Veranstaltung, selbstberichteter Lerneffekt sowie Einstellung zur Chirurgie bzw. zu chirurgischen Fachgebieten wurden mithilfe deskriptiver Analysen (Häufigkeiten, Mittelwerte, Standardabweichungen) ausgewertet. Einstellungsänderungen in Bezug auf einzelne chirurgische Fachgebiete wurden anhand des *t*-Tests für abhängige Stichproben ausgewertet.

### Stichprobe

Während der „Halleschen OP-Wochen“ wurden insgesamt 955 Prä- und 983 Post-Fragebögen ausgefüllt. In die Auswertung eingeschlossen wurden nur Medizinstudierende mit vollständig ausgefülltem Teilnehmercode und mindestens einer vollständigen Tagesevaluation (Prä- und Post-Fragebogen). Insgesamt lagen 709 vollständige Tagesevaluationen von 381 Studierenden vor, die in die vorliegende Auswertung eingehen. Mehr als 40 % der Studierenden besuchten dabei mehr als eine Operation. Tab. 1 zeigt die Anzahl der Tagesevaluationen pro Operation/Fachgebiet. Der Großteil der Studienteilnehmer/innen (60,6 %; *n* = 231) war zwischen 21 und 25 Jahren alt, 67,1 % waren weiblich, 32,9 % waren männlich. Studierende im 3. Studienjahr waren bei den „Halleschen OP-Wochen“ am häufigsten vertreten (33,5 %; *n* = 127).

## Ergebnisse

### Struktur und Organisation

Die Organisation der Veranstaltung, die Moderationen im Hörsaal und im Operationssaal, die technische Umsetzung sowie die Veranstaltung insgesamt wurden an den einzelnen Tagen von den Teilnehmenden von gut bis sehr gut bewertet. In Tab. 2 sind die Bereiche angegeben, in denen sich die Mittelwerte der einzelnen Tagesevaluationen bewegen.

**Table 2:** 

Welche Schulnote würden Sie …	MW-Bereich	SD-Bereich
der Organisation der Veranstaltung geben?	1,3–1,7	0,5–0,8
der technischen Umsetzung der Veranstaltung geben?	1,4–2,5	0,6–1,0
dem Vortragstil des Vortragenden im Hörsaal geben?	1,2–1,9	0,4–0,9
den Erläuterungen des Operateurs geben?	1,1–2,3	0,3–0,9
der Veranstaltung insgesamt geben?	1,3–1,8	0,5–0,7

Skala von 1 („sehr gut“) bis 6 („ungenügend“)

*MW* Mittelwert, *SD* Standardabweichung

### Selbstberichteter Lerneffekt

Die Teilnehmenden bewerteten die Veranstaltung überwiegend als gut bis sehr gut geeignet, den Ablauf einer Operation besser zu verstehen (Tab. 3). Besonders gut bewertet wurden die Cholezystektomie, die Tumornephrektomie, das Cochleaimplantat und die Bypassoperation. Die Teilnehmenden gaben darüber hinaus an, dass die Veranstaltung gut geeignet war, um das Verständnis für die zugrunde liegende Erkrankung zu verbessern. Bei der laparoskopischen Hysterektomie zeigten weibliche Studierende dabei eine signifikant höhere Zustimmung zur Aussage „Die Veranstaltung war geeignet, mein Verständnis der zugrunde liegenden Erkrankung zu verbessern“ als männliche Studierende (*t*(73) = −2,11; *p* < 0,05).

**Table 3:** 

*Die Veranstaltung war geeignet, mein Verständnis …*			*… für den Ablauf der Operation zu verbessern.*	*… der zugrunde liegenden Erkrankung zu verbessern.*
Fachgebiet	(*n*)	Operation	MW ± SD	MW ± SD
AC	48	Cholezystektomie	1,2 ± 0,5	2,0 ± 1,0
Ortho	63	Knieendoprothese	1,5 ± 0,6	2,2 ± 0,9
Neuro	110	Vestibularisschwannom	1,7 ± 0,9	2,0 ± 0,9
HNO	93	Cochleaimplantat	1,4 ± 0,6	2,0 ± 0,9
UC	83	Ersatzplastik vorderes Kreuzband	1,8 ± 0,9	2,1 ± 0,9
Gyn	76	Laparoskopische Hysterektomie	2,1 ± 0,9	2,5 ± 1,0
HTC	150	Bypassoperation	1,4 ± 0,6	2,3 ± 0,9
Uro	86	Tumornephrektomie	1,3 ± 0,5	2,1 ± 0,9

Skala von 1 („trifft völlig zu“) bis 5 („trifft gar nicht zu“)

*AC* Allgemeinchirurgie, *Ortho* Orthopädie, *Neuro* Neurochirurgie, *HNO* Hals-Nasen-Ohren-Heilkunde, *UC* Traumatologie, *Gyn* Gynäkologie, *HTC* Herz-Thorax-Chirurgie, *Uro* Urologie, *MW* Mittelwert, *SD* Standardabweichung

### Einstellung zu einzelnen chirurgischen Fachgebieten

Im Prä-Post-Vergleich zeigte sich, dass die Teilnahme an den Liveoperationen in einzelnen chirurgischen Fachgebieten signifikante Einstellungsänderungen bewirkte (Tab. 4). Die Bewertung der Aussage „Dieses Fachgebiet finde ich spannend“ zeigte für die Fachgebiete Hals-Nasen-Ohren-Heilkunde, Herz-Thorax-Chirurgie und Urologie signifikant positive Veränderungen.

**Table 4:** 

Fachgebiet		Spannendes Fach			Famulatur			Späteres Fachgebiet		
		*MW* *±* *SD*	*T*	*p*	*MW* *±* *SD*	*T*	*p*	*MW* *±* *SD*	*T*	*p*
AC (*n* = 48)	Prä	1,90 ± 0,79	1,35	0,18	2,30 ± 1,21	*2,21*	*<0,05*	3,35 ± 1,02	*2,46*	*<0,05*
	Post	1,83 ± 0,81			2,17 ± 1,10			3,20 ± 1,05		
Ortho (*n* = 56)	Prä	2,43 ± 1,09	1,30	0,20	2,69 ± 1,28	1,55	1,28	3,48 ± 1,26	*2,19*	*<0,05*
	Post	2,34 ± 1,12			2,56 ± 1,26			3,35 ± 1,30		
Neuro (*n* = 104)	Prä	1,81 ± 0,81	1,82	0,07	2,85 ± 1,21	*2,24*	*<0,05*	3,75 ± 1,12	*1,99*	*<0,05*
	Post	1,72 ± 0,86			2,72 ± 1,25			3,66 ± 1,19		
HNO (*n* = 90)	Prä	2,47 ± 1,13	*3,95*	*<0,001*	2,72 ± 1,30	*3,06*	*<0,01*	3,33 ± 1,34	1,06	0,29
	Post	2,22 ± 1,12			2,53 ± 1,28			3,27 ± 1,35		
UC (*n* = 78)	Prä	1,69 ± 1,02	−1,00	0,32	1,95 ± 1,18	0,00	1,00	2,91 ± 1,28	−1,27	0,21
	Post	1,74 ± 0,96			1,95 ± 1,20			2,96 ± 1,26		
Gyn (*n* = 74)	Prä	2,45 ± 1,09	−0,55	0,58	2,75 ± 1,28	−0,16	0,87	3,51 ± 1,35	0,50	0,62
	Post	2,49 ± 1,07			2,76 ± 1,35			3,48 ± 1,33		
HTC (*n* = 143)	Prä	1,50 ± 0,65	*2,91*	*<0,01*	2,22 ± 1,13	*3,14*	*<0,01*	3,18 ± 1,12	*2,52*	*<0,05*
	Post	1,41 ± 0,64			2,08 ± 1,14			3,07 ± 1,30		
Uro (*n* = 85)	Prä	2,36 ± 0,96	*3,49*	*<0,01*	2,76 ± 1,30	*3,30*	*<0,01*	3,59 ± 1,30	*3,19*	*<0,01*
	Post	2,15 ± 0,98			2,54 ± 1,27			3,39 ± 1,28		

Skala von 1 („trifft völlig zu“) bis 5 („trifft gar nicht zu“), kursiv hervorgehobene Werte zeigen signifikante Unterschiede im Prä-Post-Vergleich

*AC* Allgemeinchirurgie, *Ortho* Orthopädie, *Neuro* Neurochirurgie, *HNO* Hals-Nasen-Ohren-Heilkunde, *UC* Traumatologie, *Gyn* Gynäkologie, *HTC* Herz-Thorax-Chirurgie, *Uro* Urologie, *T* Testwert *t*-Test, *p* Signifikanzniveau, *MW* Mittelwert, *SD* Standardabweichung

In fünf der acht gezeigten Fachrichtungen (Allgemeinchirurgie, Neurochirurgie, Hals-Nasen-Ohren-Heilkunde und Urologie) zeigte sich ein signifikant positiver Effekt auf die Einstellung bezüglich des Interesses an einer Famulatur. Für die Allgemeinchirurgie, die Orthopädie, die Neurochirurgie und die Urologie zeigte sich darüber hinaus eine signifikant positive Einstellungsänderung bezüglich der potenziellen Wahl als späteres Fachgebiet.

Hinsichtlich der Einstellung der Studierenden gegenüber den Fachrichtungen zeigten sich sowohl im Prä- als auch im Post-Fragebogen für die Orthopädie und die Gynäkologie signifikante Geschlechterunterschiede. Männliche Studierende bewerteten (post) die Orthopädie als späteres Fachgebiet signifikant besser als weibliche Studierende (*t*(59) = 2,73; *p* < 0,01). Weibliche Studierende bewerteten (post) die Gynäkologie signifikant besser als männliche Studierende: spannendes Fach (*t*(80) = −3,28; *p* < 0,01), Famulatur (*t*(81) = −2,53; *p* < 0,05) und späteres Fachgebiet (*t*(79) = −3,12; *p* < 0,01). Für die anderen Fachgebiete wurden keine signifikanten Geschlechterunterschiede beobachtet.

## Diskussion

Bei den „Halleschen OP-Wochen“ wurden acht Operationen aus verschiedenen chirurgischen Fachrichtungen gezeigt, an denen insgesamt mindestens 381 Studierende der Humanmedizin fakultativ teilnahmen. Mehr als 40 % der Studierenden besuchten dabei mehr als eine Operation. Die Struktur und Organisation der Veranstaltung wurden insgesamt als (sehr) gut bewertet. Die hohen Studierendenzahlen und die gute Evaluation des Veranstaltungsformates lassen auf eine hohe Akzeptanz der Lehrveranstaltung sowie allgemein auf ein großes Interesse an der Chirurgie schließen. Der selbstberichtete Lerneffekt wurde von den Studierenden als (sehr) gut empfunden, vor allem wenn es darum ging, den Ablauf einer Operation zu begreifen. Eine Studie belegte, dass Videoübertragungen von Operationen mit paralleler Moderation Studierenden stärker die Möglichkeit bieten, eigene Fragen zu stellen sowie Fragen beantwortet zu bekommen, als die direkte Begleitung im Operationssaal [6]. Darüber hinaus wird der Lerneffekt einer solchen Veranstaltung als besser eingestuft im Vergleich zur herkömmlichen Lehre im Operationssaal [3]. Die Vermittlung ärztlicher und evidenzbasierter Handlungskompetenzen ist durch die Reform des Medizinstudiums zu kompetenzorientierten, integrierten Curricula von grundlegender Bedeutung. Liveübertragungen von Operationen im Medizinstudium stellen eine gute Möglichkeit dar, Studierenden komplexes medizinisches Arbeiten näherzubringen. Die im NKLM (Kap. 16) angeführten Lernziele, operative Eingriffe beschreiben zu können, können durch das Miterleben von Operationen möglicherweise besser erreicht werden. Durch die Kameraführung haben Studierende einen guten Einblick in das Operationsgebiet, was häufig in den Operationssälen mit einer Gruppe von Studierenden schlecht abbildbar ist. Studierende erleben das Miteinander im Team, den Umgang mit Komplikationen und werden durch die zusätzliche Moderation vertiefend über das jeweilige Krankheitsbild sowie das Operationsverfahren informiert.

Die Evaluationsergebnisse zeigen, dass die präsentierten chirurgischen Fachrichtungen insgesamt als spannend bewertet werden. Darüber hinaus sind Famulaturen für die Studierenden in den jeweiligen Fachgebieten denkbar. Die Aussage *Ich kann mir [Fachgebiet] als späteres Fachgebiet gut vorstellen erhielt bei allen Fachbereichen nur eine mittlere Zustimmung. Da fast 60 % der teilnehmenden Studierenden aus dem 1. bis 3. Studienjahr stammten, ist die Wahl des späteren Fachgebiets bei vielen wahrscheinlich noch mit großer Unsicherheit verbunden.

Das Miterleben der Liveoperation hatte einen positiven Effekt auf die Einstellung der Studierenden bezüglich der einzelnen chirurgischen Fachgebiete. Für fünf Fachgebiete konnte durch die Teilnahme an den „Halleschen OP-Wochen“ eine positive Einstellungsveränderung in Bezug auf die Wahl als späteres Fachgebiet beobachtet werden, fünf Fachgebiete wurden in der Post-Befragung als spannender bewertet und in drei Fachgebieten zeigte sich eine positive Einstellungsveränderung in Bezug auf das Interesse an einer Famulatur.

Die Teilnahme am Lehrformat ist für die Studierenden fakultativ, sodass Selektionseffekte in der Stichprobe nicht ausgeschlossen werden können. Chirurgisch interessierte Studierende sind möglicherweise insgesamt überrepräsentiert. Chirurgische Vorerfahrungen der Studierenden (z. B. durch Lehrveranstaltungen, Famulaturen/Praktika) wurden nicht erfasst, hatten sich jedoch gegebenenfalls bereits vor der Teilnahme an den „Halleschen OP-Wochen“ positiv auf die Einstellung der Studierenden zu den chirurgischen Fachgebieten ausgewirkt. Da die Studierenden größtenteils mit guten bis sehr guten Bewertung einstiegen, ist zudem eine zusätzliche Verbesserung nur begrenzt möglich.

Das übergeordnete Ziel, das Interesse von Medizinstudierenden für die Fächer der Chirurgie zu steigern, konnte durch die „Halleschen OP-Wochen“ erreicht werden. Zum einen hat die Veranstaltung „Leuchtturmcharakter“, was eine gesteigerte Aufmerksamkeit am Fach Chirurgie zur Folge hat. Zum anderen weckt das große Engagement der Chirurgen/innen und die ausführlichen Darstellungen und Erläuterungen zu operativen Abläufen das Interesse der Studierenden und motiviert ebenso, Famulaturen in einzelnen chirurgischen Fächern zu erwägen. Das breite Angebot, an verschiedenen Fachrichtungen und die Größe der Veranstaltung, d. h. die Möglichkeit mehrere Operationen anzusehen, rechtfertigt die Ausrichtung als mehrtägiges Format.

## Ausblick

Für die Studierenden der Medizinischen Fakultät an der Martin-Luther-Universität Halle-Wittenberg finden die „Halleschen OP-Wochen“ mittlerweile jährlich statt. Es werden stetig neue Fachrichtungen, wie z. B. Dermatologie, Geburtshilfe, plastische Chirurgie, Augenheilkunde, Mund-Kiefer-Gesichts-Chirurgie und seit 2016 auch Interventionen der Inneren Medizin (z. B. Endoskopie) und der interventionellen Radiologie eingebunden, wodurch die Studierenden neue typische Operationstechniken und Interventionen, Krankheitsbilder und Therapiemöglichkeiten kennenlernen. Zudem können neben den Studierenden der Human- und Zahnmedizin auch Studierende der Gesundheits- und Pflegewissenschaften sowie Schüler/innen des Ausbildungszentrums der Universitätsmedizin Halle – im Sinne einer interprofessionellen Ausbildung – an den „Halleschen OP-Wochen“ teilnehmen.

## Fazit für die Praxis

- Das Lehrformat der „Halleschen OP-Wochen“ wird von den Studierenden sehr gut angenommen und steigert das Interesse von Studierenden an einzelnen chirurgischen Fächern.
- Die Teilnahme an den „Halleschen OP-Wochen“ (u. a. durch das Miterleben von Operationen; die Moderation durch erfahrene Chirurgen/innen und die Möglichkeit, aktiv Fragen zu stellen) verbessert das Verständnis der Medizinstudierenden für chirurgische Abläufe und Operationsverfahren.
- Die Vielfalt an dargebotenen chirurgischen Interventionen bietet allen Medizinstudierenden die Möglichkeit, einen breiten Einblick in die chirurgische Tätigkeit zu bekommen.
- Die Liveübertragung von Operationen für Studierende stellt eine sinnvolle Ergänzung der chirurgischen Ausbildung dar.
